# Corrigendum to “The Efficient and Practical virus Identification System with ENhanced Sensitivity for Solids (EPISENS-S): A rapid and cost-effective SARS-CoV-2 RNA detection method for routine wastewater surveillance” [Sci. Total Environ. 843 (2022) 157101 (15 October)]

**DOI:** 10.1016/j.scitotenv.2024.176606

**Published:** 2024-12-01

**Authors:** Hiroki Ando, Ryo Iwamoto, Hiroyuki Kobayashi, Satoshi Okabe, Masaaki Kitajima

**Affiliations:** a Division of Environmental Engineering, Faculty of Engineering, Hokkaido University, North 13 West 8, Kita-ku, Sapporo, Hokkaido 060-8628, Japan; b Shionogi & Co. Ltd., 1-8, Doshomachi 3-Chome, Chuo-ku, Osaka 541-0045, Japan; c AdvanSentinel Inc., 1-8 Doshomachi 3-Chome, Chuo-ku, Osaka, Osaka 541-0045, Japan; d Research Center for Water Environment Technology, School of Engineering, The University of Tokyo, 2-11-16 Yayoi, Bunkyo-ku, Tokyo 113-0032, Japan^1^1 Present affiliation.

The authors regret that the printed version of the above article contains errors. However, these errors do not affect the findings presented in the article.

First, the authors regret an error in the description of the spiked wastewater concentrations used to compare the sensitivity between the EPISENS-S and PEG-QVR-qPCR methods throughout the manuscript, including the Graphical abstract, Abstract, Section 2.2 of the Materials and methods, Section 3.2 of the Results, and Table 1. The correct concentrations are as follows: 4.11 × 10^6^, 4.11 × 10^5^, 4.11 × 10^4^, and 4.11 × 10^3^ copies/L.

The correct Graphical abstract should appear as follows;

**Figure:**
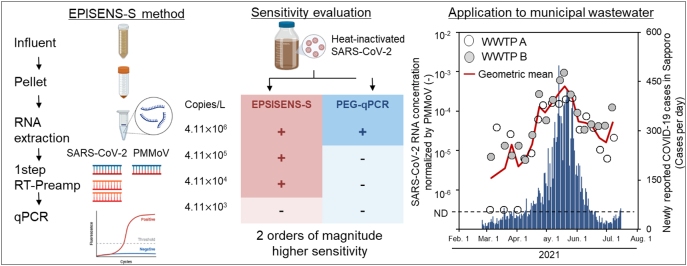

The correct Table 1 should appear as follows;

**Table 1:** Detection of SARS-CoV-2 RNA and PMMoV RNA with the EPISENS-S and PEG-qPCR methods from wastewater samples artificially contaminated with various amount of heat-inactivated SARS-CoV-2.

Method	Target	Concentration of heat-inactivated SARS-CoV-2 RNA in wastewater (copies/L)^a^							
		4.11×10^6^		4.11×10^5^		4.11×10^4^		4.11×10^3^	
		Observed concentration (copies/L)	Positive ratio	Observed concentration (copies/L)	Positive ratio	Observed concentration (copies/L)	Positive ratio	Observed concentration (copies/L)	Positive ratio
EPISENS-S	SARS-CoV-2	(4.08–4.64)×10^4^	3/3	(2.66–3.95)×10^3^	3/3	(1.75–1.02)×10^2^	3/3	N.D.	0/3
	PMMoV	(2.19–2.93)×10^7^	3/3	(2.53–19.8)×10^7^	3/3	(2.30–2.73)×10^7^	3/3	(2.41–4.45)×10^7^	3/3
PEG-qPCR	SARS-CoV-2	(1.08–3.86)×10^5^	3/3	(2.01)×10^5^	1/3	N.D.^b^	0/3	N.D.	0/3
	PMMoV	(3.30–4.55)×10^7^	3/3	(3.54–4.25)×10^7^	3/3	(4.37–8.83)×10^7^	3/3	(3.72–4.88)×10^7^	3/3

a Wastewater sample was seeded with various concentrations of heat-inactivated SARS-CoV-2 to obtain the respective final concentration.

b N.D., not determined.

Second, the authors regret that an error appears in the description of the theoretical lower limit of detection for the EPISENS-S method in the Discussion and Conclusion sections and the caption of Fig. S1. The correct value is 9.26 × 10^1^ copies/L, calculated based on the equivalent wastewater volume per reaction (i.e., 1000/10.8).

The authors would like to apologize for any inconvenience caused.

